# Differential Genetic Regulation of Canine Hip Dysplasia and Osteoarthritis

**DOI:** 10.1371/journal.pone.0013219

**Published:** 2010-10-11

**Authors:** Zhengkui Zhou, Xihui Sheng, Zhiwu Zhang, Keyan Zhao, Lan Zhu, Gang Guo, Steve G. Friedenberg, Linda S. Hunter, Wendy S. Vandenberg-Foels, William E. Hornbuckle, Ursula Krotscheck, Elizabeth Corey, Nancy S. Moise, Nathan L. Dykes, Junya Li, Shangzhong Xu, Lixin Du, Yachun Wang, Jody Sandler, Gregory M. Acland, George Lust, Rory J. Todhunter

**Affiliations:** 1 College of Animal Science and Technology, Northwest A&F University, Yangling, China; 2 Institute of Animal Science, Chinese Academy of Agricultural Sciences, Beijing, China; 3 Department of Clinical Sciences, College of Veterinary Medicine, Cornell University, Ithaca, New York, United States of America; 4 Department of Animal Science and Technology, Beijing University of Agriculture, Beijing, China; 5 Institute for Genomic Diversity, Cornell University, Ithaca, New York, United States of America; 6 Department of Computational Biology and Statistics, Cornell University, Ithaca, New York, United States of America; 7 Department of Statistics, Oklahoma State University, Stillwater, Oklahoma, United States of America; 8 Department of Animal Science, China Agricultural University, Beijing, China; 9 Guiding Eyes for the Blind, Yorktown Heights, New York, United States of America; 10 Baker Institute for Animal Health, Cornell University, Ithaca, New York, United States of America; Ohio State University Medical Center, United States of America

## Abstract

**Background:**

Canine hip dysplasia (HD) is a common polygenic trait characterized by hip malformation that results in osteoarthritis (OA). The condition in dogs is very similar to developmental dysplasia of the human hip which also leads to OA.

**Methodology/Principal Findings:**

A total of 721 dogs, including both an association and linkage population, were genotyped. The association population included 8 pure breeds (Labrador retriever, Greyhounds, German Shepherd, Newfoundland, Golden retriever, Rottweiler, Border Collie and Bernese Mountain Dog). The linkage population included Labrador retrievers, Greyhounds, and their crosses. Of these, 366 dogs were genotyped at ∼22,000 single nucleotide polymorphism (SNP) loci and a targeted screen across 8 chromosomes with ∼3,300 SNPs was performed on 551 dogs (196 dogs were common to both sets). A mixed linear model approach was used to perform an association study on this combined association and linkage population. The study identified 4 susceptibility SNPs associated with HD and 2 SNPs associated with hip OA.

**Conclusion/Significance:**

The identified SNPs included those near known genes (*PTPRD, PARD3B*, and *COL15A1*) reported to be associated with, or expressed in, OA in humans. This suggested that the canine model could provide a unique opportunity to identify genes underlying natural HD and hip OA, which are common and debilitating conditions in both dogs and humans.

## Introduction

The high relevance of canine diseases to those in humans, and the intrinsic importance of dogs to humans as special companions with a shared environment, makes the dog an attractive species to discover genes underlying the homologous, relevant diseases in humans. An important shared condition is hip dysplasia (HD) and osteoarthritis (OA), both of which are remarkably similar in their clinical expression and pathogenesis in both man and dog [1], [2], [3]. Shared phenotypic characteristics of human and canine HD are hip joint laxity accompanied by hip subluxation. The secondary effect of HD is OA which results in lameness and physical disability due to hip pain [4], [5], [6].

Canine HD, a complex trait, is a major veterinary problem occurring with a frequency up to 75% in mixed and pure breed dogs [7] of the approximately 70 million dogs in American households. Human HD, referred to as developmental dysplasia of the hip (DDH), occurs with a frequency ranging from 5∼13% [8]. Hip OA prevalence was ∼5% for individuals over 60 years old [9], [10]. Genetic predisposition and transmission models for DDH have been described [11], [12]. A locus linked to DDH spanning a 4 Mb region on HSA17q21 was recently identified [13].

Complex traits and diseases of dogs, many of which are models of homologous human conditions, are now exploitable for molecular genetic dissection [14], [15], [16], [17]. Surveys of single nucleotide polymorphisms (SNPs) within and across dog breeds reveal haplotype structures that are ideal for mapping complex traits and diseases [18]. Targeted genotyping or genome wide association studies have identified the *IGF1* gene responsible for canine body size[19], and genes associated with hair coat variation [20], degenerative myelopathy[21], and the retrogene *RFGF4* associated with the chondrodystrophoid phenotype [22].

Quantitative trait loci (QTL) influencing canine HD have shown some convergence of QTL location across studies and breeds. The challenge remains to identify the mutations that collectively underlie HD and OA in order to understand the molecular pathogenesis of these diseases and develop marker and gene based tests and novel treatments. To improve the resolution of QTL mapping for HD and OA in dogs, we extended our linkage population (Greyhound, Labrador retriever and their crosses) to include six additional breeds to reduce the extent of linkage disequilibrium (LD) between the mutation and SNPs in a genome-wide association study (GWAS). Among the 6 SNPs identified, three of them are adjacent to *COL15A1, PARD3B,* and *PTPRD* that have also been linked, or associated, with human OA. Candidate genes were suggested for the other three SNPs.

## Results

**Figure 1:**
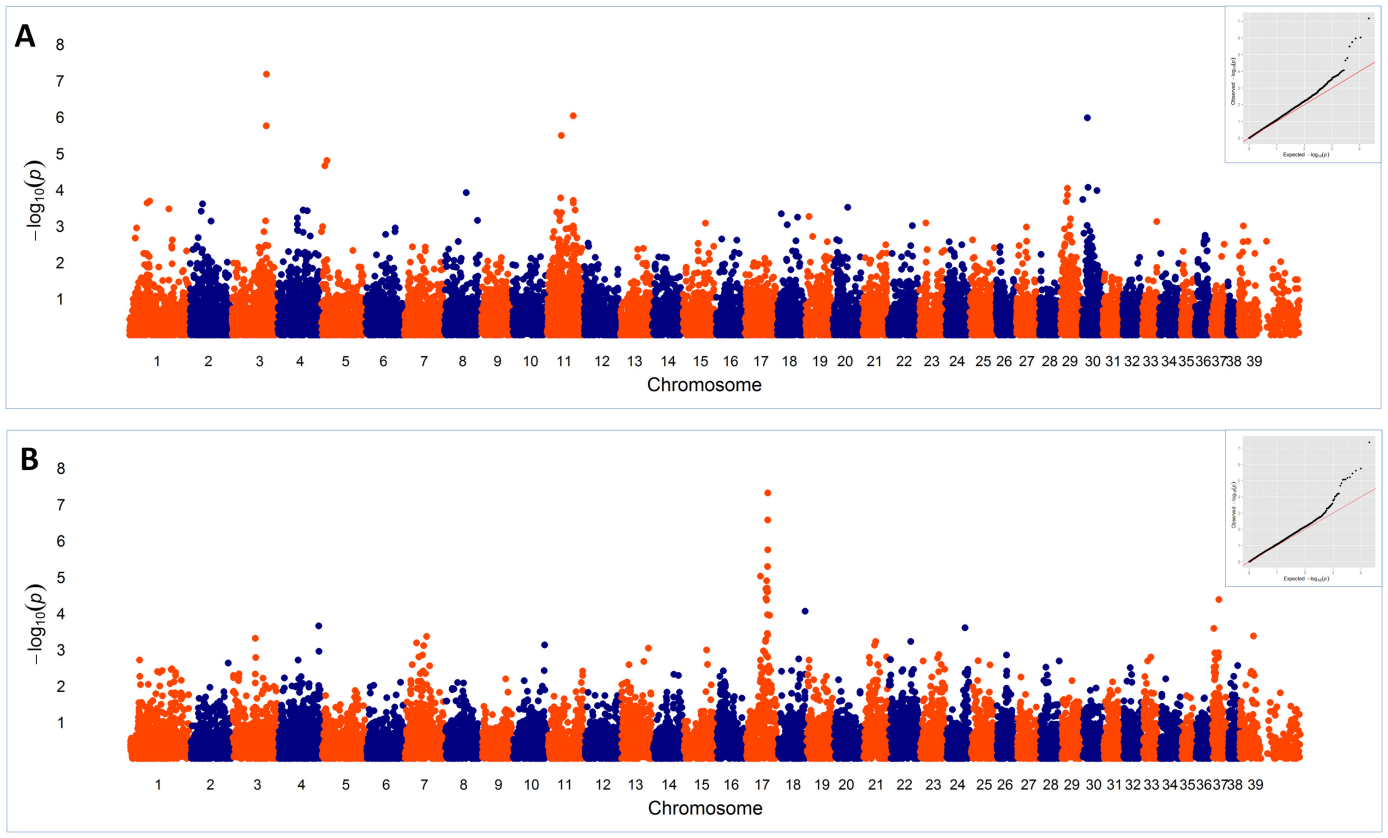
Manhattan and Quantile-Quantile (QQ) plots. The plots summarize the genome-wide association results for hip dysplasia (A) and hip osteoarthritis (B). The genome-wide *P* values (−log_10_ *P*) of the F test for the SNP effect are plotted against position on each chromosome. The inset shows QQ plots of the observed *P* values versus the expected *P* values under the null hypothesis that there was no association.

The GWAS on a linkage and association joint population identified 2 loci underlying hip OA and 4 loci underlying HD with plausible positional candidate genes (**Table 1** **and** **Figure 1**). The joint population contained 721 dogs from eight breeds and the crosses between two of the breeds (**Figure S1**). Canine HD was measured as the Norberg angle (NA) on all the breeds and crosses (**Figure S2**) and OA was measured only in Labrador retrievers, Greyhounds, and their cross breed offspring. The genotypes included 3,324 SNPs from targeted areas on 8 chromosomes (**Figure S3**) and 21,455 SNPs covering the whole genome (**Figure S4**).

**Table 1:** The associated SNPs and nearby candidate genes for hip dysplasia and osteoarthritis^a^.

SNP	Chr	Position(bp)	Allele (Risk/nonrisk)	*P* value	MAF	Distance to gene (Mb)	NotableNearby Gene
**Hip dysplasia**							
BICF2S2459425	3	74720873	G/A	6.74×10^−8^	0.354	0.900	*EVC, EVC2*
BICF2P550340	11	32935770	T/A	3.27×10^−6^	0.220	0.64	*PTPRD*
BICF2S23432143	11	57517597	G/A	9.35×10^−7^	0.394	1.577	*COL15A1*
BICF2P799261	30	13883057	C/T	1.06×10^−6^	0.135	0.109	*MAGP1*
**Osteoarthritis**							
BICF2G630205523	17	48092910	T/G	4.90×10^−8^	0.277	0.060	*REG3A*
BICF2P1242205	37	17299306	C/A	4.20×10^−5^	0.3148	0.070	*PARD3B*

a The SNPs are categorized by traits (hip dysplasia or osteoarthritis). The chromosome (Chr), position in base pair (bp), minor allele frequency (MAF), and distance to the candidate genes are listed for each SNP.

We tested the power of GWAS and the veracity of the genotype calls by mapping two known genes controlling Mendelian coat color. Among the genotyped dogs were 185 Labrador retrievers of which 101 were black, 78 were yellow, and 6 were chocolate. Black Labrador retrievers are fixed at the K locus enabling the genotype at the *MC1R* locus to be expressed [23], [24]. The *MC1R* gene causes yellow coat color in Labrador retrievers homozygous for the *MC1R* mutation. *MC1R* is located at 66.692398–66.693344 Mb on canine chromosome CFA05. When GWAS was performed on a binary trait defined as yellow versus the others (black or chocolate), SNP BICF23260560, 2 Kb away from *MC1R*, had the strongest association (*P* = 1×10^−18^). The *TYRP1* gene, located at 36.344658–36.363203 Mb on CFA11, causes chocolate coat color in black dogs [25]. When GWAS was performed on a binary trait defined as black versus chocolate, SNP BICF2P1415909, 509 Kb away from the *TYRP1* gene, showed significant association (*P* = 3×10^−7^).

For the traits of primary interest, we identified two SNPs associated with hip OA and four SNPs associated with HD in dogs. The associations were determined at the probability threshold at which the declared SNPs could be identified with a distinguishably different distribution compared to the rest of the SNPs [26] on quantile-quantile (QQ) plots. The probability thresholds were 3×10^−6^, and 5×10^−5^ for HD and hip OA, respectively.

One of the associated SNPs, at base pair 17.299306 Mb on CFA37, is near *PARD3B* which is associated with knee OA in humans [27]. However, *NRP2* lies at 17.293709–17.405724 Mb and is nearer the associated SNP locus than *PARD3B*. There may be as yet unknown genes in this interval. We identified a locus strongly associated (*P* = 4.9×10^−8^) with hip OA at 48.092910 Mb on CFA17 (**Figure 1**). The nearest known gene in the region is *REG3A* (47.930245–47.932143 Mb), 160 Kb from this locus, and which encodes a pancreatic secretory protein involved in cell proliferation or differentiation [28].

For canine HD, we discovered 4 SNPs; two of these SNPs were on CFA03 and CFA30 in which QTL for HD had been previously identified [29], [30]. and the other two SNPs were on CFA11 where QTL had also been previously identified [30], [31]. The locus at 57.517597 Mb on CFA11 is located near *COL15A1* contributing to OA in human patients [32]. The other three SNPs had no known genes associated with human HD or OA. The corresponding positional candidate genes are *EVC, EVC2* [33], *PTPRD* [34], and *MAGP1* [35].

## Discussion

Many human and canine diseases are thought to have related causes, and localizing disease genes in dogs is likely to be substantially easier than in humans [36], [37]. This is the first SNP-based, GWAS reported for canine HD and hip OA in dogs. Our goals were to resolve QTL mapping to near gene level while maintaining low risk of false positive findings (FPF) [38], [39]. In so doing, we may have overlooked some contributory loci. One of our strategies to balance the resolution and FPF was to use both ancient LD and genetic linkage retained in the crossing process. A proportion of association in our study relied on genetic linkage (recent LD) retained in the 4-generation crosses between Labrador retrievers and Greyhounds. These crosses composed 30% and 20% of the total dogs in the genome-wide screen and customized fine mapping study, respectively. The down side of the linkage population is that the extent of recent LD is much broader than ancient LD [40]. The associated SNPs we declared might be a few genes away from the causal mutations. The advantage of the linkage population is that it was used to break down the spurious associations due to population structure and cryptic relationships.

To further eliminate the potential associations due to population structure, we fitted the first 10 principal components (PCs) derived from all the SNP genotypes as covariates to capture false association due to the population structure. This sacrificed statistical power for any mutations with effects confounded with the population structure in this study. The population structure was clearly revealed by the first two PCs derived from each set of SNPs. All the dogs from within a pure breed were clustered together in the scatter plots of the first and second PCs. All the F_1_ dogs of Greyhound/Labrador retriever breedings were positioned genetically between their respective parental breeds. The backcross of the F_1_ to Labrador retriever was closer to Labrador retrievers and the backcross of F_1_ to Greyhound was genetically closer to Greyhound as expected (**Fig.** S**5**). Both sets of SNP genotypes showed substructure within the Labrador retriever breed. The substructure reflected the multiple sources of Labrador retriever for these studies; cohorts from the Guiding Eyes for Blind in Yorktown Heights NY, the Baker Institute for Animal Health at the College of Veterinary Medicine, and the dogs admitted to the Cornell University Hospital for Animals for diagnosis and treatment of lameness related to HD and hip OA.

To eliminate the spurious associations due to cryptic relationships among individual dogs, we fitted a random effect for dog groups clustered by kinship among individual dogs [41]. The kinship was derived from all the SNPs to capture unequal relatedness among dogs. This statistical model had higher statistical power than the approach that considered population structure only and the approach that considered both population structure and individual relationship without grouping [41]. The statistical power of the linkage population was 65% for a causal mutation which explained 2% of the phenotypic variation when using the linkage population only under assumption that the minor allele frequency (MAF) of the mutation is at a middle level (30%) [41].

The MAF of the SNPs from each set followed a uniform distribution (**Fig. S4**). Both sets of SNPs were informative with respective to heterozygosity. The heterozygosities showed a bimodal distribution with two peaks toward 0 and 0.5, respectively. This trend is similar to practical [42], [43], [44] and theoretical observations [45] under assumptions of the neutral theory. The mean and median heterozygosity of the 21,455 Illumina SNPs were 0.30 and 0.32, respectively. There were 4.3% SNPs with heterozygosity greater than 0.5 and 6.4% SNPs with heterozygosity less than 0.02. The mean and median heterozygosity of the customized 3,324 SNPs were 0.26 and 0.28, respectively including 2% SNPs with heterozygosity greater than 0.5 and 7.5% SNPs with heterozygosity less than 0.02.

Although the associated SNPs declared in this study had support from previous QTL linkage mapping, the current study had limited statistical power, and consequently revealed only a subset of the QTLs (10–20) reported previously [29], [30], [46], which indicated that both HD and OA were underpinned by a complex genetic model with multiple QTLs, instead of a simple genetic model for traits such as body size [47]. We previously reported HD loci through linkage analysis in a Labrador retriever/Greyhound cross breed pedigree at 63 and 64 cM on CFA11 and 18 cM on CFA30 [30]. The associated loci on CFA11 and 30 for HD in **Table 1** refine the location of the loci from linkage analysis. In our previous linkage studies for HD, we used 3 HD phenotypes including the NA and their PCs. We also used chromosome-wide, not genome-wide, permutation to establish the significance of the LOD scores. In this report, we had the most power for the NA as all the dogs had this measurement. Other loci showed possible association but we declared only those loci that had strong association signals and that had support from previous studies. Several loci linked to HD in German Shepherd Dogs were reported on CFA03 including one at ∼79 Mb [29] but these may not be identical to the CFA03 locus in our report.

Hip OA is heritable in the Labrador retriever/Greyhound cross breeds [48] and Portuguese Water Dog [46]. Linkage was found to hip OA in the Labrador retriever cross breed pedigree [49] but a significant association in the current GWAS was not discovered in the linked regions. The hip OA (acetabular osteophytes) locus reported on CFA03 in Portuguese Water Dogs was between ∼42 and 46 Mb [50] which was far from the SNP locus we reported at ∼74 Mb for HD. Similarly, although GWAS for human height only found QTLs explaining 5% of total variation, the heritability of human height was ∼  = 80%) [51].

Sample size (dogs with both genotype and phenotype), sample selection from the available population, inclusion of different dogs in the linkage studies, GWAS and fine mapping (each of which only included a subset of common dogs), marker density and distribution, and marker informativeness each both enhanced and limited the statistical power of the current study. The majority of the customized SNPs were focused on the targeted regions. A few of them were spread to cover the remainder of the LOD score profile in our previous linkage studies. The power to detect association for hip OA in our report was a limiting factor as only 99 dogs were available for this analysis and only the Labrador retrievers and the crosses to Greyhounds were included for this phenotype. Within dog breeds on average, LD is extensive (0.5–1.0 Mb), whereas across breeds, the extent of LD is less than 10 Kb [18], [19], [52]. Labrador retrievers are diversified even within breed as illustrated in **Figure S5**. The LD, on average, was less than 0.3 (R^2^) for a pair of SNPs 30 Kb and 10 Kb apart in Labrador retriever and across all breeds respectively (**Fig. S4**). The mean and median marker interval for the genome-wide SNPs was 107 Kb and 70 Kb, respectively. The mean and median marker interval for the 3,324 customized SNPs was 150 Kb and 46 Kb, respectively (**Fig. S4**). This density, in hindsight, especially for the Illumina Beadarray, was not sufficient to take full advantage of the genetic architecture in the whole genome. We expect that the current population would have more statistical power to reveal more SNPs associated with HD and hip OA if more dense markers were applied through new genotyping technology such as application of the version 2 canine Illumina Beadarray or genotyping by sequencing [53] and if more dogs were included in the GWAS.

The statistical model in which we fitted SNP genotypes across all breeds had the advantage of identifying mutations segregating in all the breeds and in the same linkage phase to the associated SNPs. However, the statistical model had low statistical power for a QTN that was in a different linkage phase from Labrador retrievers which comprised ∼50% of the individuals. An alternative mapping approach would be to treat the SNPs as confounded within breed. This statistical approach may be necessary to discover private SNPs associated with causal genes for a particular breed.

Despite the loss of statistical power in an effort to avoid false positive findings, six SNPs were identified with association. Among the positional candidate genes, *EVC* and *EVC2*, near SNPs associated with HD on CFA03, cause Ellis–van Creveld syndrome (EVC, MIM 225500) [33], a rare autosomal recessive chondrodysplasia also called chondroectodermal or mesoectodermal dysplasia. The two genes, 2.6 Kb apart, encode novel proteins with no significant homology to each other or to any other proteins. *PTPRD* (protein tyrosine phosphatase receptor type D gene), associated with HD on CFA11, encodes a member of the protein tyrosine phosphate (PTP) family. Protein tyrosine phosphatases (*PTPs*) are signaling molecules that regulate cell growth, differentiation, mitotic cycle, and oncogenic transformation. *PTPRD* belongs to the family of type IIa receptor-like protein tyrosine phosphatases[54]. It is highly expressed in human knee OA cartilage and is associated with restless legs syndrome [34], [55].

On CFA11, SNP BICF2S23432143 at 57.517597 Mb is near nicotinamide adenine dinucleotide (*NAD*) and a gene at ∼57.6 Mb identified as bA113O24.1 which is similar to IGF receptor binding proteins, based on sequence similarity. This is a positional candidate gene because *IGF1* is an anabolic growth factor for chondrocytes and *IGF*1 polymorphisms are responsible for size variation in Portuguese Water dogs and other breeds [19]. Reduced growth rate of Labrador retrievers through caloric restriction reduced phenotypic expression of HD and the accompanying hip OA [56], [57], [58]. *COL15A1*, 1.58 Mb away from SNP BICF2S23432143 on CFA11,was reported as a new positional candidate gene in genome-wide expression profiling in human OA articular cartilage[32]. *COL15A1* encodes a protein which is prominently expressed in newly-formed blood vessels indicating a possible role in angiogenesis and neovascularization. Femoral caput vascularization must occur before mineralization can proceed in the secondary center of ossification [59], [60].

An interesting positional candidate gene for HD near SNP BIC2P799261 on CFA30 is microfibril-associated protein 1 (*MFAP1*) which encodes the MFAP1 protein[61]. The microfibril associated proteins and the smaller microfibrillar associated glycoproteins appear to play roles in microfibril structure and function. Microfibrils are found in the extracellular matrices of most tissues where they serve several functional roles, including the binding and sequestration of growth factors, supplying informational signals through receptor signaling, and providing the basic structural elements for elastic fiber assembly which is important to joint capsule and ligament function. The nonglycosylated *MFAP1,* a 57 kDa protein processed to 32 kDa, was immunolocalized to microfibrils in aorta, nuchal ligament, and zonule [61]. *MFAP1* is located near *FBN1* and mutations in both genes are causal for Marfan syndrome. We are especially interested in the microfibrillary composition of the fibrous hip joint capsule as hip laxity is an important contributor, or predisposing factor, to HD. A mutation in intron 30 of *FBN2* on CFA11contributed to HD in dogs homozygous for the deletion[62]. In the cited report [62], as the degree of HD worsened in Labrador retrievers, the *FBN2* mRNA isolated from their hip capsule increased significantly consistent with developing OA. Those dogs with incipient hip OA had significantly increased *FBN2* mRNA in their hip joint capsules compared to those dogs without OA. However, dogs homozygous for the *FBN2* deletion haplotype had significantly less *FBN2* mRNA in their femoral head articular cartilage. This gene expression analysis was confounded by the incipient hip OA in HD dogs but the current data warrant further exploration for its role in HD.

SNP BICF2G630205523 on CFA17 was strongly associated with hip OA suggesting that the nearest gene *REG3A* may also play a role in canine hip OA. *REG3A* (regenerating islet derived gamma 3) was originally identified as a pancreatitis associated protein (*PAP*) released by the acini during acute pancreatitis. It is a secreted C-type lectin, reported to be upregulated in primary hepatocellular carcinomas [28]. Several functional studies demonstrated that *REG3A* may be involved in cell recognition and adhesion and in the protection of cells from oxidative stress-induced apoptosis[63]. *REG3A* possesses anti-inflammatory properties and is a signaling molecule in the *MAPK* pathway. This association offers a potential mechanism by which chondrocytes might regulate their response to injury and thereby influence the progression of hip OA.

In a GWAS for knee OA in women, SNP rs1207421, significantly associated with OA, mapped to HSA2q33(chr2:205976100) located in the *PARD3B* (par-3 partitioning defective 3 homolog B) gene [27]. In our study, its homologous gene in dog was associated with hip OA. In humans, rs1207421 is also adjacent to a previously reported linkage peak for hip OA at HSA2q31.1 in humans [64]. Moreover, this SNP was positioned in the middle of a genome-wide linkage peak mapped in extended early-onset OA families [65] making it likely that the genomic region near this marker harbors OA-susceptibility genes.

The identification of functional mutations in these candidate genes associated with canine HD or hip OA further suggest that the dog is suited for comparative mapping of homologous complex traits and diseases in humans [19], [21], [22]. No genes have yet been associated with DDH, yet positional candidate genes for HD in the dogs in our study included loci reported to be associated with human OA either through GWAS or expression studies. This raises the question of whether some of the loci associated with human hip OA contributed to a primary malformation of the hip that led to secondary hip OA. As many as 20–50% of people with hip OA in middle age had undetected DDH as neonates [9]. The earlier a developmental disease is expressed, the more likely it is to be genetic in origin assuming that primary trauma has been ruled out as a contributing factor. The secondary OA superimposed on a primary heritable defect itself affects gene expression which should be considered in microarray, transcriptome sequencing, or individual quantitative reverse transcriptase expression studies as we found in our effort to measure *FBN2* expression in hip tissues with incipient OA as a result of prior HD. Our data suggested that the rapidity of the articular response to injury seems to also be governed genetically and independently of the primary trait.

## Materials and Methods

### Dog Populations and Radiographic Methods

A total of 721 dogs from the 2,716 dogs in the HD archive [66] for this study contained a linkage population and an association population (**Figure S1** and **Table S1**). The linkage population consisted of two breeds (dysplastic Labrador retriever and non dysplastic Greyhound) and their crosses. The association population was comprised of eight breeds (Labrador retriever, Greyhound, German Shepherd, Newfoundland, Golden retriever, Rottweiler, Border Collie and Bernese Mountain Dog).

**Figure 2:**
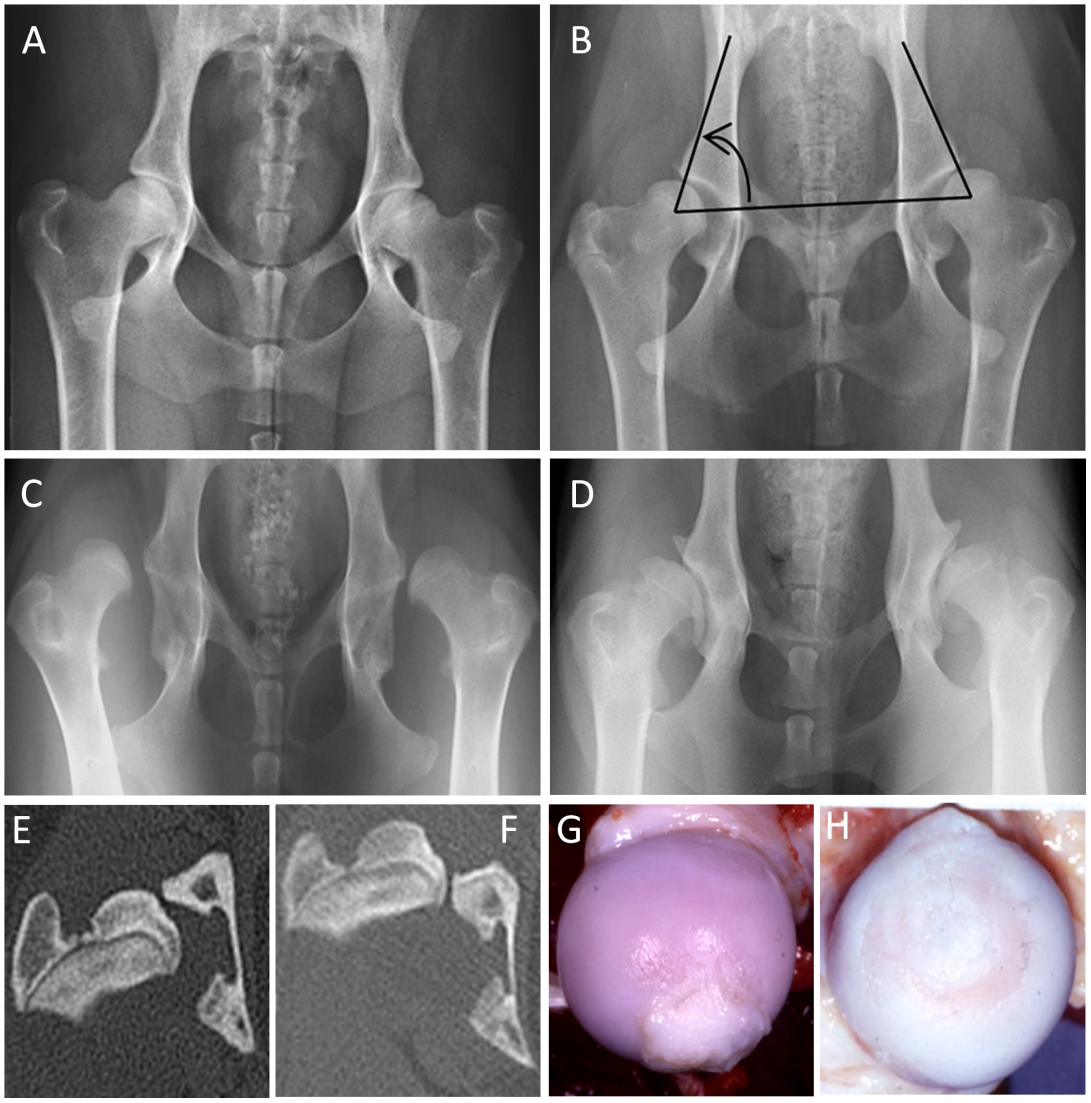
Radiographs and computed tomography (CT) images of the canine hip joint. A: Unaffected hips. B: Moderate hip dysplasia. The arrow indicates the included angle which is the Norberg angle. C: Severely affected hip dysplasia with luxation. D: Severe secondary osteoarthritis as a result of previous hip dysplasia. E: A CT image of a hip with moderate hip dysplasia illustrating the subluxation and impingement of the femoral head on the lateral acetabular rim when the dog is imaged with its hips in a weight bearing position. F: A CT image of a dog with severe hip dysplasia characterized by complete luxation. G: The gross appearance of a femoral head from a hip joint moderately affected with HD and early secondary OA with articular cartilage fibrillation in the perifoveal area and hypertrophy of the teres ligament. H: A femoral head from a severely affected hip with full thickness articular cartilage erosion and loss of the teres ligament due to mechanical abrasion and enzymatic degradation. These affected hips are painful.

The pelves of these dogs were radiographed following physical maturity (i.e. older than 8 months of age) (**Figure 2**). Hip dysplasia was measured by the Norberg angle (NA) which is a continuous trait ranging from ∼50° (a subluxated hip) to ∼120° (a phenotypically unaffected hip), see **Figure S2** and **Table S2–S3**. The NA was treated as a continuous trait in the statistical modeling. The worst hip (the minimum) between measurements on left and right from each dog was used in the statistical analyses.

The presence or absence of hip OA was determined on dysplastic Labrador retrievers and their crosses to unaffected Greyhounds. Hip OA was defined radiographically, as osteophytes on the acetabular rim and periphery of the femoral head and neck or at autopsy, as fibrillation and erosion of the articular cartilage of the femoral head and acetabulum, increased synovial fluid volume, and hypertrophy of the teres ligament and fibrous joint capsule [67]. We used a synovial fluid volume >0.3 ml, a displacement volume of the round ligament of the femoral head >0.8 ml, and a perifoveal articular cartilage lesion of fibrillation or ulceration as incontrovertible evidence of the presence of hip OA. Hip OA was defined in two ways. The majority of dogs used in the analyses for hip OA had postmortem evidence of OA which was defined above and by Lust et al. [67], [68]. A small portion of dogs were scored from the radiographic evidence of hip OA based on the OFA hip score assigned by a board certified radiologist. Dogs with any acetabular osteophytes, osteophytes around the femoral head or enthesiophytes on the femoral neck were determined to have hip OA [69]. Based on these gross observations, we then assigned dogs affected with hip OA as cases and unaffected as controls. Of the 99 dogs evaluated for hip OA, 50 were unaffected (controls) and 49 had OA (cases) (**Table S4**). The coat color was recorded for the 185 Labrador retriever dogs. Cornell Institutional Animal Care and Use Committee approved all of the studies (Protocol approval numbers were 2005-0151 (Cornell Medical Genetic Archive) and 2006-0187 (Hip Dysplasia and OA Genetics).

### Selection of Dogs and SNP Genotyping

The 721 dogs were genotyped on two different SNP platforms. One was the Illumina CanineSNP20 Bead array containing ∼ 22,000 loci distributed across the entire canine genome. The other array was customized with ∼3,500 SNPs chosen from the regions in eight chromosomes (2, 3, 5, 11, 18, 19, 29, and 30) harboring QTLs for HD and some for hip OA identified in previous studies [30], [49], [70]. The SNPs for custom genotyping were chosen from the CanFam 2.0 database provided by the Broad Institute at Harvard/MIT (http://www.broadinstitute.org/science/projects/mammals-models/dog/dog-snps-canfam-20). The custom SNPs were chosen to maximize the number of Labrador retriever origin SNPs and intragenic SNPs. The physical order of the custom SNPs selected were alternated by breed to span the QTL linkage interval defined by the LOD score profile (personal communication, Kerstin Lindblad-Toh, 2007). The QTL intervals for chromosome 05, 18, and 19, were linked to hip OA [49] while those on chromosomes 02, 03, 11, 29 and 30 were linked to HD based on microsatellite screening [70]. The SNPs for genotyping were chosen to be denser under the LOD score linkage peak and less dense on either side of the estimated peak LOD score position (**Figure. S3**).

We selected one SNP every ∼45 Kb in the following locations on the canine genome: CFA03, between 55–75 Mb; CFA11, between 4–31 and 38–65 Mb; CFA 29, between 9–31 Mb; CFA30, between 13–39 Mb. Each of these chromosomes including CFA11 and 29 harbored at least 1 QTL contributing to canine HD. In aggregate, these regions comprise 122 Mb. From the Broad canine SNP data base, the most valid SNPs (those identified by capitalization) were chosen. Where ever possible, we included SNPs derived from Labrador retrievers as Labrador retrievers or their crosses comprised the most common breeds among our genotypes dogs. We attempted to select at least one SNP within the boundaries of each gene in our target regions. Additionally, among the 902 genes in our target regions, we selected SNPs within 534 of the 902 genes in our target regions ; the remainder had no intragenic SNPs in the Broad database.

In total, we selected 2,799 SNPs across the four chromosomes described above. By breed, these SNPs represent: 31% boxer, 26% poodle, 5% Labrador retriever (enriched from 1% in the Broad database), 4% German shepherd, and the remainder miscellaneous breeds ( e.g., Bedlington terrier, Portuguese water dog, Akita).

To fine map the hip OA locus, we selected 592 SNPs on 4 chromosomes at 4 loci: CFA02 between 10–60 Mb, CFA05 between 40–70 Mb, CFA18 between 3–30 Mb, and CFA19 between 3–15 Mb. These correspond to regions where our microsatellite screen showed association with hip OA. On average, we selected 1 SNP every 181 Kb. These SNPs arose from 13% Labradors, 11% Boxers, 8% Malamutes, 8% Portuguese water dogs, and 60% other breeds. Of the 861 genes in these regions, we had SNPs within 377 genes or 44% of them. The SNPs were selected from the Broad Harvard/MIT web site. The customized genotyping used Molecular Inversion Probe Technology [71].

There were 366 and 551 dogs genotyped with the Illumina array and the customized array, respectively. Genotypes were obtained on 196 dogs across both arrays. All the genotyped dogs had at least one phenotype for HD. However, only 99 dogs were phenotyped for OA. The 48 dogs with OA phenotype that were genotyped on the customized array were included in the dogs that were genotyped on the Illumina array (**Table S1, S2, S3**). DNA was extracted from whole blood collected in Na-EDTA using either phenol-chloroform or the Puregene (Gentra) protocol [30]. Physical locations of candidate genes were based on the UCSC web browser (http://genome.ucsc.edu/cgi-bin/hgGateway).

The majority (92.3%) of the Illumina SNPs had call rate of 100%. There was 99.46% of SNPs with call rate above 95%. For the customized SNP array, 84.9% of SNPs had a call rate above 95%. SNPs with missing calls above 45% were removed. We also removed SNPs with minor allele frequency (MAF) below 1% [72]. The final analysis contained 21,455 SNPs for the Illumina array and 3,324 SNPs for the customized array. For the Illumina SNP array, the mean and median MAF were 0.2394 and 0.2399, respectively. For the customized SNP array, the mean and median MAF were 0.2205 and 0.2110, respectively (**Fig. S4**). There were 111 SNPs in common between the two sets of SNP arrarys. The concordance rate was 99.999% of the 111 common SNPs genotyped on 196 dogs.

### Statistical analysis

A compressed mixed linear model was used for the association analysis. This approach is superior to other approaches with respect to minimizing false positive finding due to population structure and cryptic relationships accompanied by increased statistical power [41]. The fixed effects in the model included sex, age and the first ten principal components (PCs) derived from all the SNPs. The PCs were calculated from the numerical genotypes coded as 0 or 2 for the two homozygote classes, or 1 for the heterozygote class. Because the first two PCs captured the breed structure (**Figure S5**), we did not include breed as a co-factor in the model. The random effect was the groups clustered based on the kinship among individual dogs. The kinship among individuals was derived from all the SNPs. Individuals were clustered into groups which contained 3 individual dogs on average based on the optimization from our previous study [41].

The genotype of each SNP was treated as a fixed effect, one SNP at a time. The *P* values resulting from the *F* test were used to indicate the association strength. The majority of the SNPs from the two sets were analyzed independently. The 111 common SNPs underwent a joint analysis by combining all 721 dogs together. For the 196 dogs genotyped with both SNP sets, there were two sets of PCs and kinships. We used the SNPs from the Illumina array in the joint analysis. As the PCs derived from the two DNA arrays do not exactly reflect the same genetic components, we fitted PC nested within the two SNP sets. For the gap in kinship between the dogs genotyped on only one of the arrays, we used their pedigree based kinship. We used the visual approach [26] to determine whether there was an excess of significant associations by comparing the *P* values for the association tests to those expected under the null distribution using Quantile-Quantile (QQ) plots.

## Supporting Information

Figure S1 The linkage and association joint population.
(0.08 MB PDF)

Figure S2 Distribution of phenotyped dogs on Norberg Angle measurements.
(0.03 MB PDF)

Figure S3 The chromosome coverage of the customized SNP array.
(0.03 MB PDF)

Figure S4 The properties of single nucleotide polymorphisms (SNPs)
(0.06 MB PDF)

Figure S5 Population structure of linkage and association joint population.
(0.18 MB PDF)

Table S1 Number of dogs categorized by breed, phenotype and SNP platforms.
(0.01 MB PDF)

Table S2 Distribution of Norberg Angle measurements (number of dogs).
(0.01 MB PDF)

Table S3 Number of dogs with Norberg Angle measurements categorized by study population and SNP array.
(0.01 MB PDF)

Table S4 Total number of dogs with hip osteoarthritis _OA_ records categorized by study population and SNP array.
(0.01 MB PDF)
