# Supplementary material for: Differential Genetic Regulation of Canine Hip Dysplasia and Osteoarthritis
Source: PLoS One. 2010 Oct 11;5(10):e13219. doi: 10.1371/journal.pone.0013219 (PMC2952589; doi:10.1371/journal.pone.0013219)
Supplement: Figure S1 — The linkage and association joint population. (0.08 MB PDF) [file pone.0013219.s001.pdf]

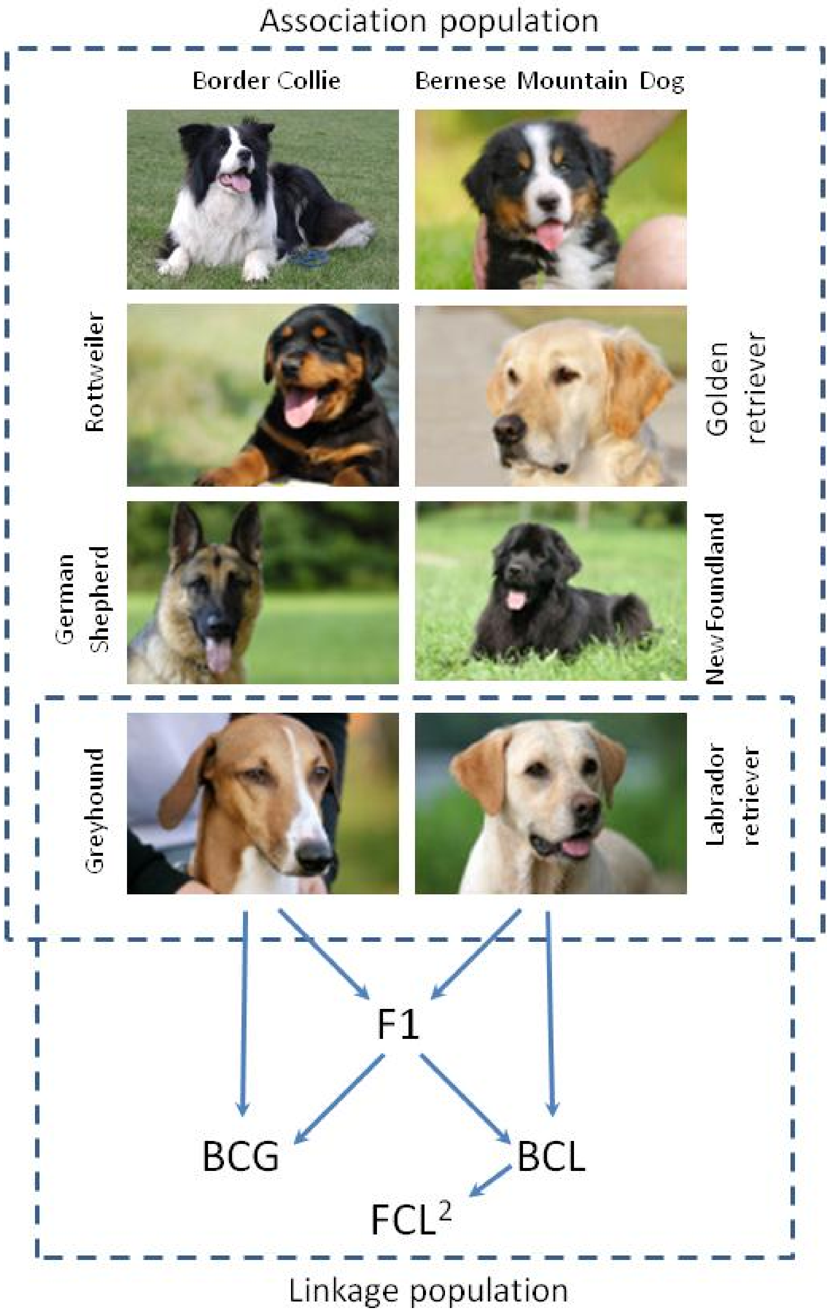

Figure S1. The linkage and association joint population. The association population (8 pure breeds) contributed ancient linkage disequilibrium and the linkage population (2 breeds and their crosses) contributed recent linkage disequilibrium for mapping quantitative trait loci. F1= LR X G cross. BCG=F1 backcross to Greyhound, BCL=F1 backcross to Labrador retriever. F2=cross between F1s, The images are credited to the contributor from Flickr (http://www.flickr.com).
