# Supplementary material for: Differential Genetic Regulation of Canine Hip Dysplasia and Osteoarthritis
Source: PLoS One. 2010 Oct 11;5(10):e13219. doi: 10.1371/journal.pone.0013219 (PMC2952589; doi:10.1371/journal.pone.0013219)
Supplement: Figure S2 — Distribution of phenotyped dogs on Norberg Angle measurements. (0.03 MB PDF) [file pone.0013219.s002.pdf]

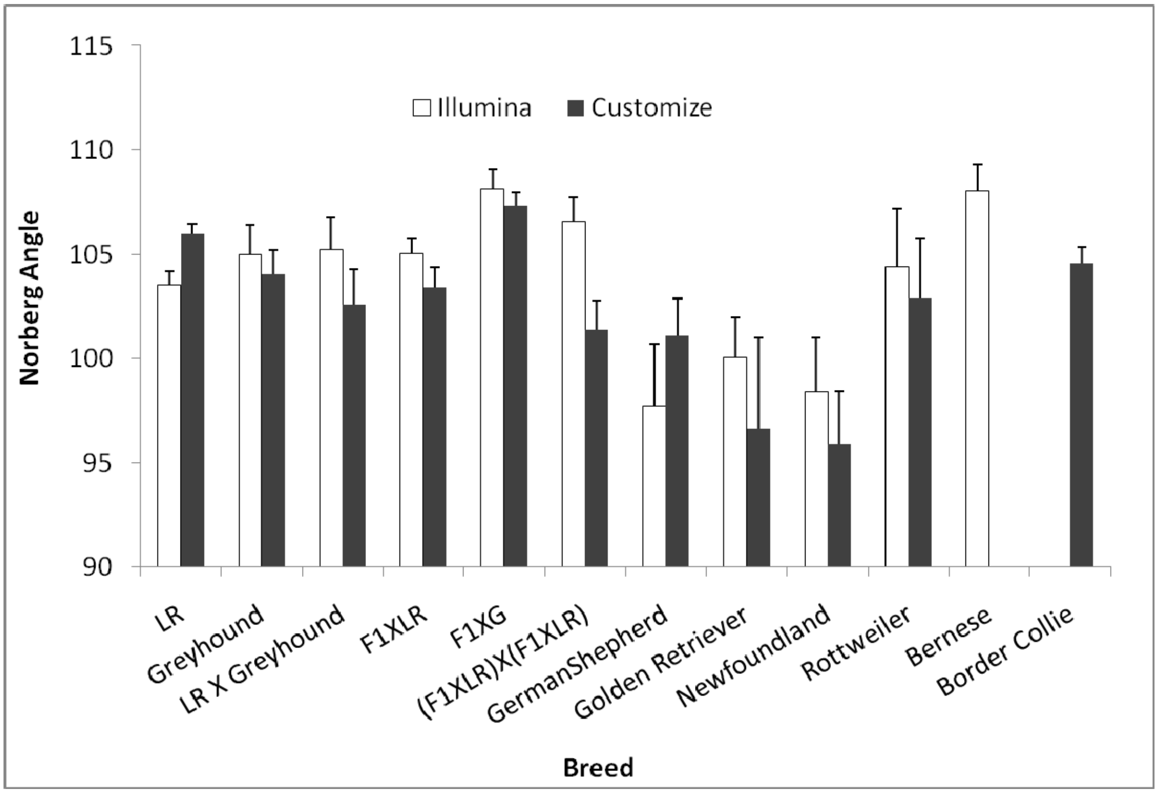

Figure S2. Distribution of phenotyped dogs on Norberg Angle measurements. The distribution was categorized by breed for the Illumina and customized SNP array. Canine hip dysplasia was measured as Norberg Angle on left and right sides. The worst hip (the minimum) measurement - between left and right hips from each dog was used in the analyses. The mean and standard error of the worst hip measurement are displayed for dogs genotyped with the Illumina and customized SNP array, respectively. LR=Labrador retriever, F1= LR X Greyhound cross, G= Greyhound.
