# Supplementary material for: Differential Genetic Regulation of Canine Hip Dysplasia and Osteoarthritis
Source: PLoS One. 2010 Oct 11;5(10):e13219. doi: 10.1371/journal.pone.0013219 (PMC2952589; doi:10.1371/journal.pone.0013219)
Supplement: Figure S3 — The chromosome coverage of the customized SNP array. (0.03 MB PDF) [file pone.0013219.s003.pdf]

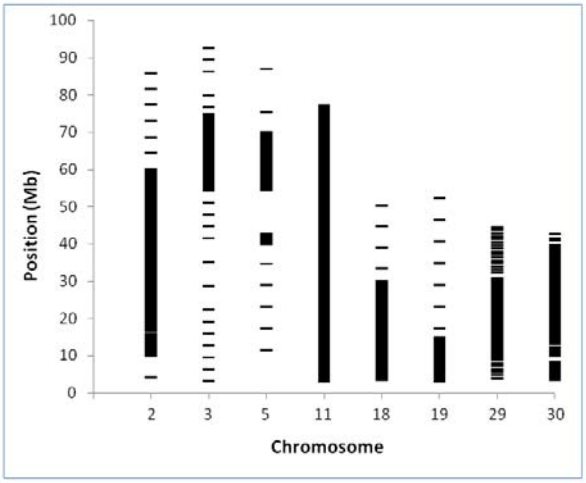

Figure S3. The chromosome coverage of the customized SNP array. The array contained ~3,300 informative SNPs on 8 targeted chromosomes that harbored the quantitative trait loci for HD and some for hip osteoarthritis identified from the previous studies. The SNPs for custom genotyping were chosen from the Broad Institute at Harvard/MIT.
