# Supplementary material for: Differential Genetic Regulation of Canine Hip Dysplasia and Osteoarthritis
Source: PLoS One. 2010 Oct 11;5(10):e13219. doi: 10.1371/journal.pone.0013219 (PMC2952589; doi:10.1371/journal.pone.0013219)
Supplement: Figure S4 — The properties of single nucleotide polymorphisms (SNPs) (0.06 MB PDF) [file pone.0013219.s004.pdf]

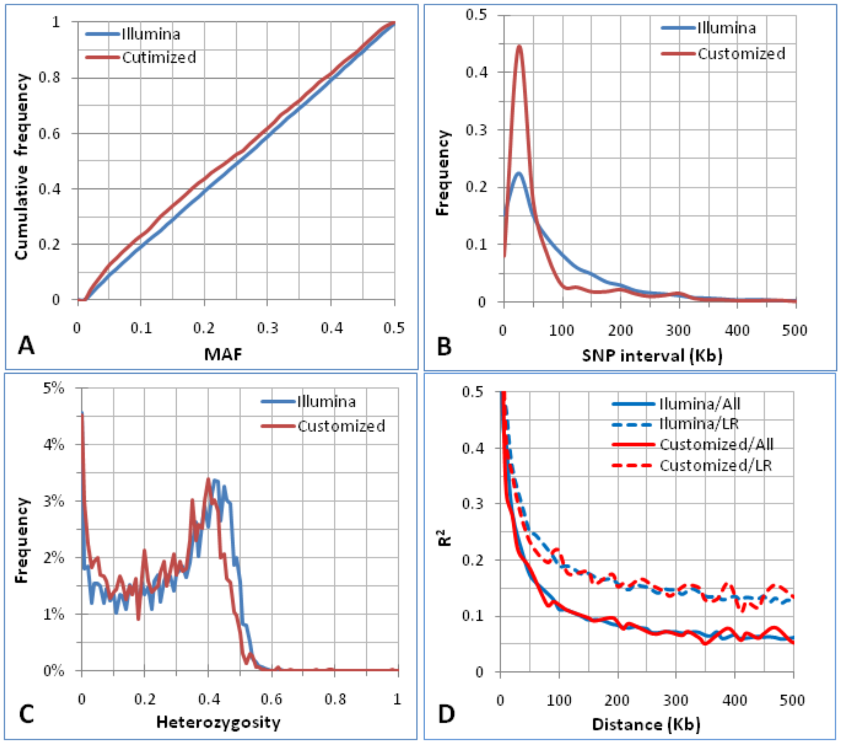

Figure S4. The properties of single nucleotide polymorphisms (SNPs). Dogs were genotyped with customized and Illumina arrays. A: Cumulative distribution of minor allele frequencies B: The density of the filtered SNPs; C: Distribution of heterozygosity; D: Linkage disequilibrium (LD) decay (R^2) over physical distance. The LD was calculated with all breeds and all Labrador Retrievers (LR) respectively.
