# Supplementary material for: Differential Genetic Regulation of Canine Hip Dysplasia and Osteoarthritis
Source: PLoS One. 2010 Oct 11;5(10):e13219. doi: 10.1371/journal.pone.0013219 (PMC2952589; doi:10.1371/journal.pone.0013219)
Supplement: Figure S5 — Population structure of linkage and association joint population. (0.18 MB PDF) [file pone.0013219.s005.pdf]

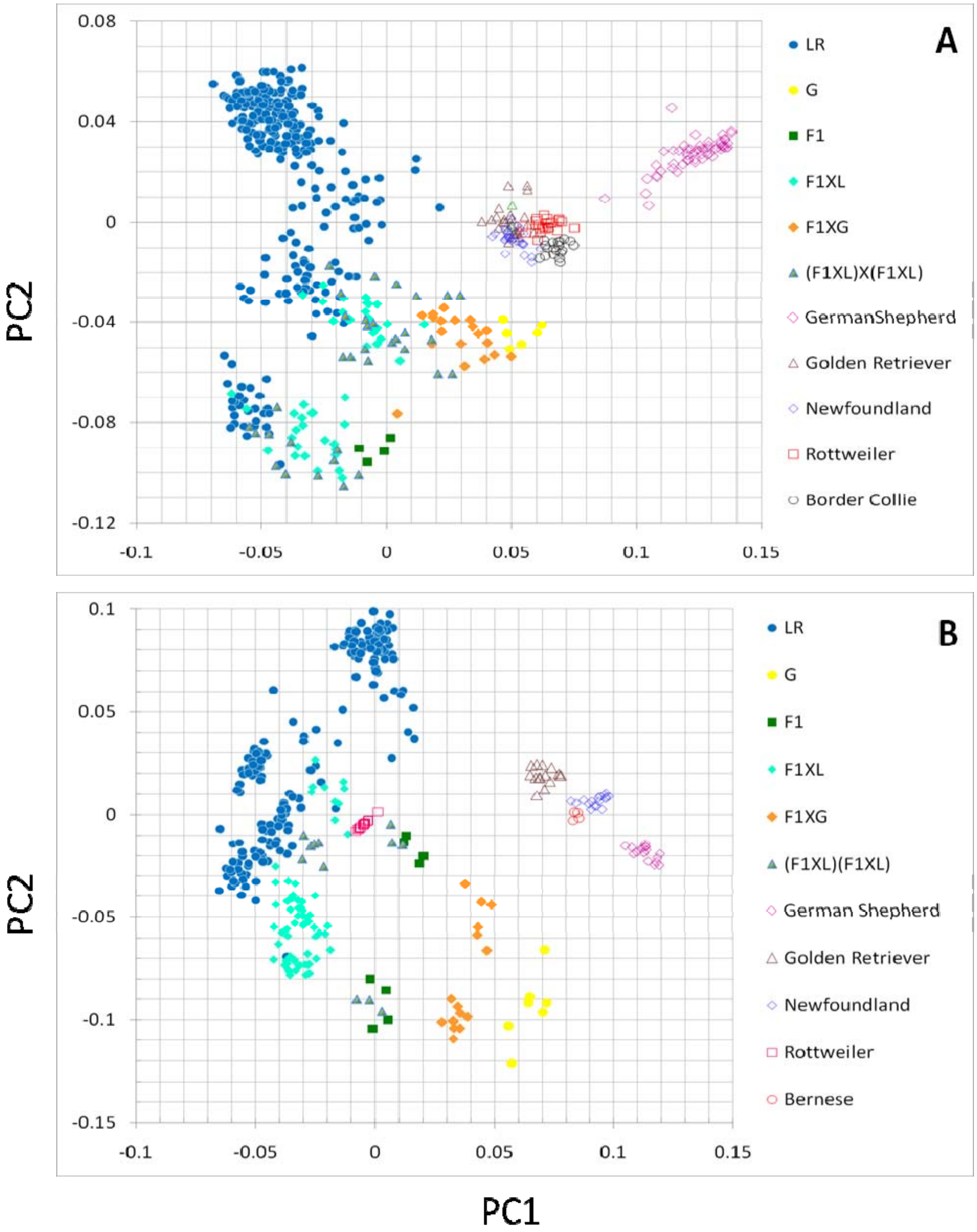

Figure S5. Population structure of linkage and association joint population. The population structure was characterized by first and second principal components (PC). The PCs were derived from all single nucleotide polymorphisms (SNPs) from the customized (A) and Illumina arrays (B). L=Labrador retriever (LR), G=Greyhound, F1= LR X G cross.
