# Supplementary material for: Differential Genetic Regulation of Canine Hip Dysplasia and Osteoarthritis
Source: PLoS One. 2010 Oct 11;5(10):e13219. doi: 10.1371/journal.pone.0013219 (PMC2952589; doi:10.1371/journal.pone.0013219)
Supplement: Table S1 — Number of dogs categorized by breed, phenotype and SNP platforms. (0.01 MB PDF) [file pone.0013219.s006.pdf]

Table S1. Number of dogs categorized by breed, phenotype and SNP platforms.

Breed	Hip dysplasia Illumina SNP array	Hip dysplasia Customized SNP array	Hip dysplasia Overlap	Osteoarthritis Illumina SNP array	Osteoarthritis Customized SNP array	Osteoarthritis Overlap
Labrador retriever	188	288	85	42	14	14
Greyhound	7	6	6	-	-	-
LR × Greyhound (F1)	8	5	4	1		
F1×LR	67	47	41	37	21	21
F1×Greyhound	16	19	8	12	7	7
(F1×LR) × (F1×LR)	12	34	11	7	6	6
Golden retriever	15	26	2	-	-	-
German Shepherd	17	56	10	-	-	-
Newfoundland	18	28	16	-	-	-
Rottweiler	14	23	13	-	-	-
Bernese Mt. Dog	4	-	-	-	-	-
Border Collie	-	19	-	-	-	-
Total	366	551	196	99	48	48

There were 721 (366+551-196) dogs genotyped with Illumina array, customized array or both (overlap). Each of the 721 dogs had a Hip dysplasia measurement (Norberg angle). Of these 721 dogs, 99 dogs had measurements on hip Osteoarthritis. LR= Labrador retriever, F1=Labrador retriever crossed with Greyhound.
