# Supplementary material for: Differential Genetic Regulation of Canine Hip Dysplasia and Osteoarthritis
Source: PLoS One. 2010 Oct 11;5(10):e13219. doi: 10.1371/journal.pone.0013219 (PMC2952589; doi:10.1371/journal.pone.0013219)
Supplement: Table S2 — Distribution of Norberg Angle measurements (number of dogs). (0.01 MB PDF) [file pone.0013219.s007.pdf]

Table S2. Distribution of Norberg Angle measurements (number of dogs).

Norberg Angle (º)	Illumina Array	Customized array	Overlap	Total
~70	2	8	2	8
71~80	7	19	6	20
81~90	17	22	12	27
91~100	64	69	43	90
101~110	226	337	114	449
111~120	50	96	19	127
Total	366	551	196	721

There were721 dogs genotyped with the Illumina array, the customized array, or both (overlap). Each dog had a measurement on Norberg Angle which is a continuous trait ranging from ~50º (a subluxated hip) to ~120º (a phenotypically unaffected hip).
