# Supplementary material for: Differential Genetic Regulation of Canine Hip Dysplasia and Osteoarthritis
Source: PLoS One. 2010 Oct 11;5(10):e13219. doi: 10.1371/journal.pone.0013219 (PMC2952589; doi:10.1371/journal.pone.0013219)
Supplement: Table S3 — Number of dogs with Norberg Angle measurements categorized by study population and SNP array. (0.01 MB PDF) [file pone.0013219.s008.pdf]

Table S3. Number of dogs with Norberg Angle measurements categorized by study population and SNP array.

Population	Illumina array	Customized array	Overlap	Total
Linkage	298	399	155	542
Association	263	446	132	577
Overlap	195	294	91	398
Total	366	551	196	721

There were721 dogs genotyped with the Illumina array, the customized array or both (overlap). These dogs were from two populations (linkage and association). The linkage population included Labrador retrievers, Greyhounds and their crosses. The association population included eight purebreeds (Labrador retrievers, Greyhounds, German Shepherds, Newfoundlands, Golden retrievers, Rottweilers, Border Collies and Bernese Mountain Dogs).
