# Supplementary material for: Differential Genetic Regulation of Canine Hip Dysplasia and Osteoarthritis
Source: PLoS One. 2010 Oct 11;5(10):e13219. doi: 10.1371/journal.pone.0013219 (PMC2952589; doi:10.1371/journal.pone.0013219)
Supplement: Table S4 — Total number of dogs with hip osteoarthritis _OA_ records categorized by study population and SNP array. (0.01 MB PDF) [file pone.0013219.s009.pdf]

**Table S4.** Total number of dogs with hip osteoarthritis (OA) records categorized by study population and SNP array.

| Population | Illumina array | Customized array | Overlap | Total |
|---|---|---|---|---|
| Linkage | 99(49) | 48(14) | 48(14) | 99(49) |
| Association | 42(22) | 14(10) | 14(10) | 42(22) |
| Overlap | 42(22) | 14(10) | 14(10) | 42(22) |
| Total | 99(49) | 48(14) | 48(14) | 99(49) |

There were 99 dogs with hip osteoarthritis measurements. The numbers in parenthesis are the number of dogs with osteoarthritis (cases). These 99 dogs were genotyped with either Illumina array, the customized array or both (overlap). These dogs were from two populations (linkage and association). The linkage population included Labrador retrievers, Greyhounds and their crosses. The association population included eight purebreeds (Labrador retrievers, Greyhounds, German Shepherds, Newfoundlands, Golden retrievers, Rottweilers, Border Collies and Bernese Mountain Dogs)
